# Doubling Time of the COVID-19 Epidemic by Province, China

**DOI:** 10.3201/eid2608.200219

**Published:** 2020-08

**Authors:** Kamalich Muniz-Rodriguez, Gerardo Chowell, Chi-Hin Cheung, Dongyu Jia, Po-Ying Lai, Yiseul Lee, Manyun Liu, Sylvia K. Ofori, Kimberlyn M. Roosa, Lone Simonsen, Cecile Viboud, Isaac Chun-Hai Fung

**Affiliations:** Georgia Southern University, Statesboro, Georgia, USA (K. Muniz-Rodriguez, D. Jia, M. Liu, S.K. Ofori, I.C.-H. Fung);; Georgia State University, Atlanta, Georgia, USA (G. Chowell, Y. Lee, K.M. Roosa);; National Institutes of Health, Bethesda, Maryland, USA (G. Chowell, C. Viboud); Independent researcher, Hong Kong (C.-H. Cheung);; Boston University, Boston, Massachusetts, USA (P.-Y. Lai);; Roskilde University, Roskilde, Denmark (L. Simonsen)

**Keywords:** China, coronavirus, COVID-19, infectious disease transmission, epidemiology, respiratory infections, viruses, 2019 novel coronavirus disease, SARS-CoV-2, severe acute respiratory syndrome coronavirus 2, viruses, zoonoses

## Abstract

In China, the doubling time of the coronavirus disease epidemic by province increased during January 20–February 9, 2020. Doubling time estimates ranged from 1.4 (95% CI 1.2–2.0) days for Hunan Province to 3.1 (95% CI 2.1–4.8) days for Xinjiang Province. The estimate for Hubei Province was 2.5 (95% CI 2.4–2.6) days.

Our ability to estimate the basic reproduction number (R_0_) of emerging infectious diseases is often hindered by the paucity of information about the epidemiologic characteristics and transmission mechanisms of new pathogens (*1*). Alternative metrics could synthesize real-time information about the extent to which the epidemic is expanding over time. Such metrics would be particularly useful if they rely on minimal and routinely collected data that capture the trajectory of an outbreak (*2*).

Epidemic doubling times characterize the sequence of intervals at which the cumulative incidence doubles (*3*). If an epidemic is growing exponentially with a constant growth rate *r*, the doubling time remains constant and equals (ln 2)/*r*. An increase in the doubling time indicates a slowdown in transmission if the underlying reporting rate remains unchanged (Appendix) (*4*).

We analyzed, by province, the number of times coronavirus disease (COVID-19) cumulative incidence doubled and the evolution of the doubling times in mainland China (*5*), from January 20 (when nationwide reporting began) through February 9, 2020. We retrieved province-level daily cumulative incidence data from provincial health commissions’ websites and conducted 2 sensitivity analyses based on a longer and a shorter time period (Appendix). We excluded Tibet from further analysis because only 1 case was reported during the study period.

During January 20–February 9, the harmonic mean of the arithmetic means of the doubling times estimated from cumulative incidence ranged from 1.4 (95% CI 1.2–2.0) days in Hunan Province to 3.1 (95% CI 2.1–4.8) days in Xinjiang Province. We estimated doubling time as 2.5 (95% CI 2.4–2.6) days in Hubei Province. The cumulative incidence doubled 6 times in Hubei Province during the study period. The harmonic mean of the arithmetic means of doubling times for mainland China except Hubei Province was 1.8 (95% CI 1.5–2.3) days. Fujian, Guangxi, Hebei, Heilongjiang, Henan, Hunan, Jiangxi, Shandong, Sichuan, and Zhejiang provinces had a harmonic mean of the arithmetic means of doubling times <2 days (Figure; Appendix Figure 1).

**Figure:**
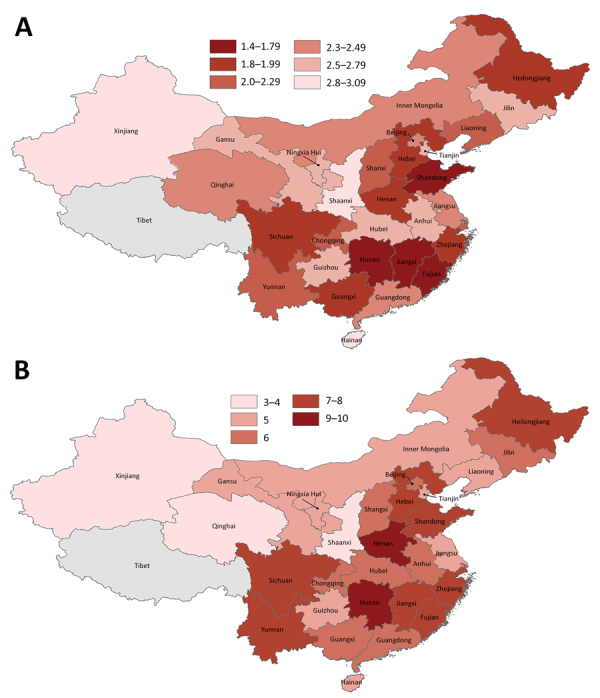
Doubling time estimates for coronavirus disease in mainland China, by province, January 20–February 9, 2020. A) Harmonic mean of the arithmetic means of doubling time estimates; B) number of times the cumulative incidence doubled during the study period.

As the epidemic progressed, it took longer for the cumulative incidence in mainland China (except Hubei) to double, which indicated an overall subexponential growth pattern outside Hubei Province (Appendix Figures 1, 2). In Hubei Province, the doubling time decreased and then increased. A gradual increase in the doubling time coincided with the social distancing measures and intraprovincial and interprovincial travel restrictions imposed across China since the implementation of the quarantine of Wuhan on January 23 (*6*).

Our estimates of doubling times are shorter than prior estimates. Li et al. covered cases reported by January 22 and found a doubling time estimate of 7.4 (95% CI 4.2–14) days (*5*). Wu et al. statistically inferred case counts in Wuhan by internationally exported cases as of January 25 and estimated doubling time as 6.4 (95% CI 5.8–7.1) days (*7*). Volz et al. identified a common viral ancestor on December 8, 2019, using Bayesian phylogenetic analysis and fitted an exponential growth model to provide the epidemic growth rate and estimated a doubling time of 7.1 (95% CI 3.0–20.5) days (*8*). Our estimates are based on cumulative confirmed case count by reporting date by province during January 20–February 9, 2020.

Our study is subject to several limitations, including underreporting of cases (*9*). One reason for underreporting is underdiagnosis, resulting from a lack of diagnostic tests, healthcare workers, and other resources. Further, underreporting is likely heterogeneous across provinces. As long as reporting remains invariant over time within the same province, the calculation of doubling times remains reliable; however, this is a strong assumption. Growing awareness of the epidemic and increasing availability of diagnostic tests might have strengthened reporting over time, which could have artificially shortened the doubling time. Nevertheless, apart from Hubei and Guangdong Provinces (first cases reported on January 19, 2020), nationwide reporting began only on January 20; at that point, authorities in China openly acknowledged the magnitude and severity of the epidemic. 

Because of a lack of detailed case data describing incidence trends for imported and local cases, we focused our analysis on the overall trajectory of the epidemic without adjusting for the role of imported cases on the local transmission dynamics. It is likely that the proportion of imported cases could be large for provinces that reported only a few cases; their short doubling times in the study period could simply reflect rapid detection of imported cases. However, with the data through February 9, only 2 provinces had a cumulative case count <40 (Appendix Table 1). It would be worthwhile to investigate the evolution of the doubling time after accounting for case importations if more detailed data become available.

In summary, we observed an increasing trend in the epidemic doubling time of COVID-19 by province of China during January 20–February 9, 2020. The harmonic mean of the arithmetic means of doubling times of cumulative incidence during the study period in Hubei Province, where the outbreak was first recognized, was estimated at 2.5 (95% CI 2.4–2.6) days.

Appendix Additional information on the study of doubling time of the COVID-19 epidemic in China.
