# Supplementary material for: Doubling Time of the COVID-19 Epidemic by Province, China
Source: Emerg Infect Dis. 2020 Aug;26(8):1912–4. doi: 10.3201/eid2608.200219 (PMC7392464; doi:10.3201/eid2608.200219)
Supplement: Appendix — Additional information on the study of doubling time of the COVID-19 epidemic in China. [file 20-0219-Techapp-s1.pdf]

Article DOI: https://doi.org/10.3201/eid2608.200219

Doubling Time of the COVID-19 Epidemic by Province, China

Appendix

Motivation, Scope, and Methods

Motivation

Reproduction number (R_0), a widely used indicator of transmission potential in a totally susceptible population, is driven by the average contact rate and the mean infectious period of the disease (*1*). However, it characterizes only transmission potential at the onset of the epidemic and varies geographically for a given infectious disease according to local healthcare provision, outbreak response, and socioeconomic and cultural factors. Furthermore, estimating R_0 requires information about the natural history of the infectious disease. Thus, our ability to estimate reproduction numbers for novel infectious diseases is hindered by the paucity of information about their epidemiologic characteristics and transmission mechanisms. More informative metrics could synthesize real-time information about the extent to which the epidemic is expanding over time. Such metrics would be particularly useful if they rely on minimal data on the outbreak's trajectory (*2*).

Scope and Definitions

Our analysis in this article is restricted to mainland China. A "province" encompasses 3 different types of political subdivisions of mainland China: a province, a centrally (literally, "directly") administered municipality (Beijing, Chongqing, Shanghai, and Tianjin), and an "ethnic minority" autonomous region (Guangxi, Inner Mongolia, Ningxia, Tibet, and Xinjiang). Our analysis does not include the Hong Kong Special Administrative Region and the Macau Special Administrative Region, which are under the effective rule of the People's Republic of China through the "One Country, Two Systems" political arrangements. Our analysis also does not include Taiwan, which is governed de facto by a different government (the Republic of China).

Data Sources

Daily cumulative incidence data were retrieved from provincial health commissions' websites (Appendix Table 8). Data were double-checked against the cumulative national total published by the National Health Commission (*3*), data compiled by the Centre for Health Protection, Hong Kong, when available (*4*), and data from John Hopkins University Center for Systems Science and Engineering (*5*). Whenever discrepancies arose, provincial government sources were deemed authoritative.

Doubling Time Calculation and Relationship with Epidemic Growth Rate

As the epidemic grows, the times at which cumulative incidence doubles are given by t_{d_i} such that $2C(t_{d_i}) = C(t_{d_{i+1}})$, where $t_{d_o} = 0$, $C(t_{d_o}) = C_0$, and i = 0,1,2,3, …, n_d where n_d is the total number of times cumulative incidence doubles. The actual sequence of doubling times is defined as follows:

$$d_j = \Delta t_{d_j} = t_{d_j} - t_{d_{j-1}} \text{ where } j = 1,2,3, \ldots, n_d.$$

To quantify parameter uncertainty, we used parametric bootstrapping with a Poisson error structure around the harmonic mean of doubling times d_j to obtain the 95% confidence interval (*6*–*8*).

If we assume homogeneous mixing (equal probability of acquiring infection through contacts) and exponential growth, then $C(t_2) = C(t_1)\exp(rt)$; therefore, $\ln(C(t_2)/C(t_1)) = rt$. When $C(t_2)/C(t_1) = 2$, t is the doubling time; that is, $t = t_d$, $\ln 2 = rt_d$. Therefore, the doubling time, t_d, equals $(\ln 2)/r$ (*9*).

Methods

We calculated doubling time using MATLAB R2019b (Mathworks, https://www.mathworks.com). We created the figures using either R version 3.6.2 (R Core Team, https://www.r-project.org) or MATLAB R2019b. Significance level in this manuscript was a priori decided to be $\alpha = 0.05$.

Results and Discussion

Cumulative Incidence over Time

Appendix Figures 7–10 provide plots of cumulative incidence over time (left panels) and semilog plots with log_{10}-transformed cumulative incidence over time (right panels) for 8 provinces with a relatively high number of cases: the epicenter, Hubei, followed by (in alphabetical order) Fujian, Guangdong, Heilongjiang, Henan, Hubei, Hunan, Jiangxi, and Shandong. If the epidemic is growing exponentially, the log_{10}-transformed cumulative incidence over time will be a linear curve. If social distancing would have an impact, the slope of the semilog plot would decrease, indicating a decreasing epidemic growth rate.

Harmonic Mean of the Harmonic Mean

In this study, we also presented the harmonic mean of the harmonic means of the estimates of the epidemic doubling times. The harmonic means of the epidemic doubling times are shorter than their arithmetic means. During January 20–February 9, 2020, the harmonic mean of the harmonic means of the doubling times estimated ranged from 0.5 days (95% CI 0.2–1.3) for Guangxi to 2.3 days (95% CI 2.3–2.4) for Hubei. The harmonic mean of the harmonic means of doubling times in mainland China except Hubei were 1.2 days (95% CI, 1.0–1.4) (Appendix Table 4).

Further Discussion

The slowing down of the epidemic as represented in increasing epidemic doubling times in our study is also consistent with a study by Benjamin F. Maier and Dirk Brockmann, “Effective containment explains sub-exponential growth in confirmed cases of recent COVID-19 outbreak in Mainland China” (preprint available at arXiv 2020:2002.07572). They also identified subexponential growth of the outbreak across provinces, as mass quarantine and restriction of travels across mainland China began, since January 23, 2020.

Sensitivity Analysis 1

We performed a sensitivity analysis by expanding our data analysis to the data after December 31, 2019, when Hubei first reported a cluster of pneumonia cases with unexplained etiology that turned out to be COVID-19. The only difference between the sensitivity analysis and the main analysis is the inclusion of Hubei and Guangdong data from December 31, 2019, through January 19, 2020, because nationwide reporting started on January 20, 2020. The only

differences in results were found for Hubei and Guangdong. For Hubei, the harmonic mean of the arithmetic mean of the doubling times was 4.06 (95% CI 3.85–4.33), the harmonic mean of the harmonic means of the doubling times for Hubei was 2.28 (95% CI 2.08–2.56), and the cumulative incidence in Hubei doubled nine times from December 31, 2019, through February 9, 2020 (Appendix Table 5, Appendix Figures 3, 4, 12–14). The first doubling time of Hubei (Appendix Figure 3) was high, reflecting that real-time data were unavailable before mid-January. It was only from January 17, 2020 onward that data reporting become increasingly transparent and timely.

Sensitivity Analysis 2

We also performed a sensitivity analysis by restricting our data analysis to the data for January 23–February 9, 2020, to allow for the time that all the other provinces to ramp up their testing. January 23 was also the day when the Chinese authorities to put the city of Wuhan on lockdown and major interprovincial travel restrictions were put in place. When we changed the start date of our study period from January 20 (main analysis) to January 23, 2020 (sensitivity analysis 2), the epidemic doubling time of the aggregate cumulative incidence of mainland China (except Hubei) increased from 1.79 (95% CI 1.52–2.25) to 2.90 (95% CI 2.62–3.24) (harmonic mean of the arithmetic means), and from 1.18 (95% CI 0.96–1.42) to 1.98 (95% CI 1.82–2.17) (harmonic mean of the harmonic means) (Appendix Table 7, Appendix Figures 5, 6). Apart from the epidemic doubling time of the aggregate cumulative incidence of mainland China (except Hubei), we did not observe significant differences by province between results in the main analysis and sensitivity analysis 2. Therefore, our results should be robust for the purpose of this study.

References

1. Anderson RM, May RM. Infectious diseases of humans. Oxford: Oxford University Press; 1991.

2. Drake JM, Bakach I, Just MR, O'Regan SM, Gambhir M, Fung IC-H. Transmission models of historical Ebola outbreaks. Emerg Infect Dis. 2015;21:1447–50. PubMed https://doi.org/10.3201/eid2108.141613

3. National Health Commission of the People's Republic of China. 2020 [cited 2020 Feb 2]. http://www.nhc.gov.cn/

4. Centre for Health Protection, Department of Health, The Government for the Hong Kong Special Administrative Region. 2020 [cited 2020 Feb 2]. https://www.chp.gov.hk/en/index.html

5. John Hopkins University Center for Systems Science and Engineering. 2019 Novel Coronavirus COVID-19 (2019-nCoV) Data Repository by Johns Hopkins CSSE. 2020 [cited 2020 Feb 13]. https://github.com/CSSEGISandData/COVID-19

6. Banks HT, Hu S, Thompson WC. Modeling and inverse problems in the presence of uncertainty. Boca Raton (FL): CRC Press; 2014.

7. Chowell G, Ammon CE, Hengartner NW, Hyman JM. Transmission dynamics of the great influenza pandemic of 1918 in Geneva, Switzerland: Assessing the effects of hypothetical interventions. J Theor Biol. 2006;241:193–204. PubMed https://doi.org/10.1016/j.jtbi.2005.11.026

8. Chowell G, Shim E, Brauer F, Diaz-Dueñas P, Hyman JM, Castillo-Chavez C. Modelling the transmission dynamics of acute haemorrhagic conjunctivitis: application to the 2003 outbreak in Mexico. Stat Med. 2006;25:1840–57. PubMed https://doi.org/10.1002/sim.2352

9. Vynnycky E, White RG. An Introduction to Infectious Disease Modelling. Oxford: Oxford University Press; 2010.

Appendix Table 1. Confirmed cases of COVID-19 (December 31, 2019–January 19, 2020) by province in mainland China extracted from official government sources used for the sensitivity analysis.*

	Dec	January																		
Locations†	31	1	2	3	4	5	6	7	8	9	10	11	12	13	14	15	16	17	18	19
Mainland China (excluding Hubei) (sum of provincial reports)	NR	NR	NR	NR	NR	NR	NR	NR	NR	NR	NR	NR	NR	NR	NR	NR	NR	NR	NR	1
Mainland China (including Hubei) (sum of provincial reports)	27	NR	NR	44	NR	59	NR	NR	NR	NR	41	41	41	41	41	41	45	62	121	199
Mainland China (including Hubei) (sum by NHC)‡	NA	NA	NA	NA	NA	NA	NA	NA	NA	NA	NA	NA	NA	NA	NA	NA	NA	NA	NA	NA
Hubei	27	NR	NR	44	NR	59	NR	NR	NR	NR	41	41	41	41	41	41	45	62	121	198
Guangdong	NR	NR	NR	NR	NR	NR	NR	NR	NR	NR	NR	NR	NR	NR	NR	NR	NR	NR	NR	1

*NA, not applicable; NHC, National Health Commission of China; NR, not reported.
†Observations were collected directly from government official sites from each province in mainland China. If a press release included data reported at midnight and early morning, they were considered to belong to the day before the data were reported.
‡Official national tally of cumulative case count of confirmed cases was first published by the National Health Commission of China (NHC) on January 21, 2020 for January 20, 2020 (*3*).

Appendix Table 2. Confirmed cases of COVID-19 (January 20–31, 2020) by province in mainland China; data extracted from official government sources used for the main analysis and sensitivity analysis.

	January											
Locations*	20	21	22	23	24	25	26	27	28	29	30	31
Mainland China (excluding Hubei) (sum of provincial reports)	26	71	145	291	585	923	1321	1802	2386	3126	3885	4637
Mainland China (including Hubei) (sum of provincial reports)	296	446	589	840	1314	1975	2744	4516	5940	7712	9691	11790
Mainland China (including Hubei) (sum by NHC)†	291	440	571	830	1287	1975	2744	4515	5974	7711	9692	11791
Hubei	270	375	444	549	729	1052	1423	2714	3554	4586	5806	7153
Anhui	0	1	9	15	39	60	70	106	152	200	237	297
Beijing	5	10	14	26	36	49	68	80	91	111	132	156
Chongqing	0	5	9	27	57	75	110	132	147	165	206	238
Fujian	0	0	1	5	10	18	35	59	82	101	120	144
Gansu	0	0	0	2	4	7	14	19	24	26	29	35
Guangdong	14	26	32	53	78	98	146	188	241	311	393	520
Guangxi	0	0	2	13	23	33	46	51	58	78	87	100
Guizhou	0	0	0	3	5	5	7	9	9	12	15	29
Hainan	0	0	4	8	11	20	27	33	40	46	49	57
Hebei	0	0	1	2	8	13	18	33	48	65	82	96
Heilongjiang	0	0	1	4	9	15	21	30	37	43	59	80
Henan	0	1	5	9	32	83	128	168	206	278	352	422
Hunan	0	1	4	9	43	69	100	143	221	277	332	389
Inner Mongolia	0	0	0	1	2	7	11	13	16	18	20	23
Jiangsu	0	0	1	9	18	31	47	70	99	129	168	202
Jiangxi	0	2	3	7	18	36	48	72	109	162	240	286
Jilin	0	0	1	3	4	4	6	8	9	14	14	17
Liaoning	0	0	2	4	12	19	22	30	36	41	45	60
Ningxia	0	0	1	2	3	4	7	11	12	17	21	26
Qinghai	0	0	0	0	0	1	4	6	6	6	8	9
Shaanxi	0	0	0	0	7	15	22	46	56	63	87	101
Shandong	0	1	1	1	21	39	63	87	87	145	178	202
Shanghai	2	9	16	20	33	40	53	66	80	101	128	153
Shanxi	0	0	1	1	6	9	13	20	27	35	39	47
Sichuan	0	2	5	15	28	44	69	90	108	142	177	207
Tianjin	0	2	4	5	8	10	14	23	25	27	32	32
Tibet	0	0	0	0	0	0	0	0	0	1	1	1
Xinjiang	0	0	0	2	3	4	5	10	13	14	17	18
Yunnan	0	1	1	2	5	11	19	26	51	70	80	91
Zhejiang	5	10	27	43	62	104	128	173	296	428	537	599

*Observations were collected directly from government official sites from each province in mainland China. If a press release included data reported at midnight and early morning, they were considered to belong to the day before the data were reported. NHC, National Health Commission of China.
†Data were collected from NHC press releases (*3*).

Appendix Table 3. Confirmed cases of COVID-19 (February 1–9, 2020) by province in mainland China, extracted from official government sources used for the main analysis and sensitivity analysis.

	February								
Locations*	1	2	3	4	5	6	7	8	9
Mainland China (excluding Hubei) (sum of provincial reports)	5396	6031	6910	7646	8352	9049	9614	10098	10507
Mainland China (including Hubei) (sum of provincial reports)	14381	17208	20432	24324	28017	31161	34567	37198	40138
Mainland China (including Hubei) (sum by NCH)†	14380	17205	20438	24324	28018	31161	34546	37198	40171
Hubei	9074	11177	13522	16678	19665	22112	24953	27100	29631
Anhui	340	408	480	530	591	665	733	779	830
Beijing	183	212	228	253	274	297	315	326	337
Chongqing	262	300	337	366	389	411	426	446	468
Fujian	159	179	194	205	215	224	239	250	261
Gansu	40	51	55	57	62	67	71	79	83
Guangdong	604	683	797	870	944	1018	1075	1120	1131
Guangxi	111	127	139	150	168	172	183	195	210
Guizhou	38	46	56	64	69	77	89	96	99
Hainan	63	70	79	89	100	111	122	128	136
Hebei	104	113	126	135	157	171	195	206	218
Heilongjiang	95	118	155	190	227	277	295	307	331
Henan	493	566	675	764	851	914	981	1033	1073
Hunan	463	521	593	661	711	772	803	838	879
Inner Mongolia	27	34	35	42	46	50	52	54	58
Jiangsu	236	271	308	341	373	408	439	468	492
Jiangxi	333	391	476	548	600	661	698	740	771
Jilin	23	31	42	54	59	65	69	78	80
Liaoning	64	73	74	81	89	94	99	105	108
Ningxia	28	31	34	34	40	43	45	45	49
Qinghai	9	13	15	17	18	18	18	18	18
Shaanxi	116	128	142	165	173	184	195	208	213
Shandong	225	246	270	298	343	379	407	435	466
Shanghai	177	193	208	233	254	269	281	292	295
Shanxi	56	66	74	81	90	96	104	115	119
Sichuan	231	254	282	301	321	344	363	386	405
Tianjin	45	48	60	67	69	81	88	90	94
Tibet	1	1	1	1	1	1	1	1	1
Xinjiang	21	24	29	32	36	39	42	45	49
Yunnan	99	109	117	122	128	135	138	140	141
Zhejiang	661	724	829	895	954	1006	1048	1075	1092

*Observations were collected directly from government official sites from each province in mainland China. If a press release included data reported at midnight and early morning, they were considered to belong to the day before the data were reported. NHC, National Health Commission of China.
†Data were collected from NHC press releases (*3*).

Appendix Table 4. Main analysis: Doubling times of COVID-19 cumulative incidence and their harmonic mean of the arithmetic means of the doubling times and harmonic mean of the harmonic means of the doubling times (95% Confidence interval) by province in mainland China, January 20–February 9, 2020.

Category		Mainland China (Except Hubei)	Hubei	Anhui	Beijing	Chongqing	Fujian	Gansu	Guangdong	Guangxi	Guizhou	Hainan
Harmonic mean of arithmetic means		1.79 (1.52–2.25)	2.54 (2.44–2.64)	2.56 (2.16–3.11)	2.49 (1.89–3.38)	2.22 (1.53–3.22)	1.71 (1.15–2.52)	2.56 (2.00–3.78)	2.47 (1.97–3.20)	1.92 (1.45–3.09)	2.71 (1.90–3.90)	2.91 (1.91–3.89)
Harmonic mean of harmonic means		1.18 (0.96–1.42)	2.34 (2.27–2.41)	1.72 (1.13–2.67)	1.48 (0.63–2.70)	1.23 (0.67–1.96)	0.82 (0.46–1.41)	1.36 (0.76–2.86)	2.01 (1.53–2.54)	0.48 (0.22–1.34)	1.88 (0.81–3.28)	1.52 (0.65–2.99)
Times doubled	1	0.59	2.91	2.12	1.00	2.05	0.25	1	1.33	0.18	2.5	1.00
	2	0.86	2.16	0.75	1.5	0.56	0.5	1.14	1.79	0.36	3.5	1.55
	3	0.98	1.5	2.18	1.8	0.82	0.85	1.26	2.17	0.76	1.64	2.28
	4	1	2.17	1.77	2.7	1.71	1.15	4.1	2.38	1.6	2.56	5.31
	5	1.3	3.03	4.03	4.14	3.58	1.07	5.9	2.76	3.4	5.8	6.86
	6	1.98	3.43	4.9	7.31	4.82	1.39		4.92	4.78		
	7	2.55					3.12					
	8	4.53					9.21					

Category		Hebei	Heilongjiang	Henan	Hunan	Inner Mongolia	Jiangsu	Jiangxi	Jilin	Liaoning	Ningxia	Qinghai
Harmonic mean of arithmetic means		1.88 (1.57–2.72)	1.93 (1.76–2.36)	1.81 (1.35–2.05)	1.42 (1.24–2.04)	2.37 (1.80–3.67)	2.43 (1.77–3.26)	1.68 (1.45–2.33)	2.64 (2.13–3.50)	2.10 (1.45–3.30)	2.54 (1.76–4.33)	2.50 (1.50–5.00)
Harmonic mean doubling time		1.04 (0.67–1.93)	1.08 (0.62–1.98)	0.81 (0.56–1.12)	0.71 (0.47–1.13)	1.17 (0.67–2.67)	1.93 (1.35–2.63)	1.13 (0.71–1.71)	1.48 (0.66–3.03)	1.05 (0.54–1.94)	1.59 (0.73–3.07)	1.00 (0.38–3.87)
Times doubled	1	1	0.33	0.25	0.33	1	2	1.25	0.5	1	1	0.33
	2	0.33	0.67	0.5	0.67	0.4	1.31	0.84	2.5	0.5	2	0.67
	3	0.67	0.8	1	0.8	0.85	1.75	0.72	2	1.07	1.25	4
	4	1.6	1.36	0.55	0.4	2.75	2.32	0.96	3.66	2.76	2.55	4.5
	5	1.33	2.12	0.7	0.47	4.71	4.07	1.89	2.43	4.67	4.53	
	6	2.01	2.95	0.62	1.13			1.69	3.74			
	7	5.28	3.04	1.38	1.85			1.99				
	8		3.31	2.69	1.97			4.16				
	9			3.57	4.22							
	10			6.56								

Category		Shaanxi	Shandong	Shanghai	Shanxi	Sichuan	Tianjin	Tibet	Xinjiang	Yunnan	Zhejiang
Harmonic mean of the arithmetic means		2.82 (2.12–9.97)	1.68 (1.42–2.39)	2.19 (1.88–2.68)	2.31 (1.67–3.25)	1.83 (1.39–2.70)	2.78 (2.07–4.06)	Not applied	3.05 (2.06–4.75)	2.05 (1.34–2.72)	1.91 (1.60–2.51)
Harmonic mean doubling time		2.04 (1.28–3.01)	0.48 (0.28–1.15)	0.77 (0.34–1.73)	1.22 (0.68–2.51)	0.96 (0.51–1.75)	1.69 (0.80–3.55)	Not applied	1.91 (0.83–4.46)	1.25 (0.89–1.81)	1.20 (0.74–1.70)
Times doubled	1	1.33	2.05	0.28	1.2	0.66	1		2	2	1
	2	2.24	0.1	0.57	0.4	0.64	2		1.6	0.66	0.58
	3	3.76	0.19	1.15	1.06	0.77	2.22		3.06	0.84	1.23
	4		0.41	1.92	1.76	1.18	4.78		5.34	1.12	1.61
	5		0.86	2.92	2.2	1.55	3.57			1.61	2.29
	6		1.43	3.16	4.18	2.78				1.45	1.47
	7		2.66	6.13		4.49				7.32	3.48
	8		4.71								

Appendix Table 5. Sensitivity analysis 1 (continued in Appendix Table 6): doubling times of COVID-19 cumulative incidence and their harmonic mean of the arithmetic means of the doubling times and harmonic mean of the harmonic means of the doubling times (95% CI) by province in mainland China, December 31, 2019–February 9, 2020: mainland China (except Hubei), Hubei, and from Anhui to Qinghai.

Category		Mainland China (except Hubei)	Hubei	Anhui	Beijing	Chongqing	Fujian	Gansu	Guangdong	Guangxi	Guizhou	Hainan
Harmonic mean of arithmetic means		1.34 (1.28–1.52)	4.06 (3.85–4.33)	2.57 (2.12–3.00)	2.51 (1.99–3.26)	2.22 (1.60–3.23)	1.82 (1.18–2.55)	2.55 (1.83–3.79)	1.88 (1.74–2.19)	1.93 (1.47–2.96)	2.78 (2.00–3.97)	2.92 (1.97–4.25)
Harmonic mean of harmonic means		0.29 (0.15–0.59)	2.28 (2.08–2.56)	1.76 (1.21–2.40)	1.60 (0.93–2.70)	1.23 (0.74–1.88)	0.83 (0.47–1.42)	1.33 (0.70–2.62)	0.44 (0.25–1.13)	0.49 (0.22–1.29)	1.98 (1.09–3.53)	1.55 (0.60–3.29)
Times doubled	1	0.04	17.33	2.12	1.00	2.05	0.25	1.00	0.07	0.18	2.50	1.00
	2	0.08	1.22	0.75	1.5	0.56	0.5	1.14	0.16	0.36	3.5	1.55
	3	0.15	2	2.18	1.8	0.82	0.85	1.26	0.3	0.76	1.64	2.28
	4	0.33	3.04	1.77	2.7	1.71	1.15	4.1	0.63	1.6	2.56	5.31
	5	0.53	2.11	4.03	4.14	3.58	1.07	5.9	1.84	3.4	5.8	6.86
	6	0.73	1.23	4.9	7.31	4.82	1.39		1.44	4.78		
	7	0.92	2.61				3.12		2.18			
	8	0.98	3.13				9.21		2.59			
	9	1.01	3.87						2.72			
	10	1.48							6.17			
	11	2.17										
	12	2.88										
	13	5.55										
		Hebei	Heilongjiang	Henan	Hunan	Inner Mongolia	Jiangsu	Jiangxi	Jilin	Liaoning	Ningxia	Qinghai
Harmonic mean of arithmetic means		1.89 (1.55–2.74)	1.96 (1.76–2.26)	1.80 (1.31–2.10)	1.41 (1.26–1.99)	2.37 (1.82–3.57)	2.45 (1.75–3.31)	1.72 (1.44–2.36)	2.67 (2.13–3.50)	2.16 (1.49–3.53)	2.58 (1.72–4.43)	2.64 (1.79–5.00)
Harmonic mean of harmonic means		1.07 (0.66–1.90)	1.12 (0.66–1.97)	0.77 (0.48–1.14)	0.73 (0.48–1.15)	1.15 (0.65–2.71)	1.92 (1.31–2.68)	1.17 (0.81–1.74)	1.60 (0.70–3.11)	1.06 (0.50–2.45)	1.67 (0.87–3.65)	0.96 (0.39–3.69)
Times doubled	1	1.00	0.33	0.25	0.33	1.00	2.00	1.25	0.50	1.00	1.00	0.33
	2	0.33	0.67	0.5	0.67	0.4	1.31	0.84	2.5	0.5	2	0.67
	3	0.67	0.8	1	0.8	0.85	1.75	0.72	2	1.07	1.25	4
	4	1.6	1.36	0.55	0.4	2.75	2.32	0.96	3.66	2.76	2.55	4.5
	5	1.33	2.12	0.7	0.47	4.71	4.07	1.89	2.43	4.67	4.53	
	6	2.01	2.95	0.62	1.13			1.69	3.74			
	7	5.28	3.04	1.38	1.85			1.99				
	8		3.31	2.69	1.97			4.16				
	9			3.57	4.22							
	10			6.56								

Appendix Table 6. Sensitivity analysis 1 (continued from Appendix Table 5): doubling times of COVID-19 cumulative incidence and their harmonic mean of the arithmetic means of the doubling times and harmonic mean of the harmonic means of the doubling times (95% Confidence interval) by province in mainland China, December 31, 2019–February 9, 2020: from Shaanxi to Zhejiang.

Category		Shaanxi	Shandong	Shanghai	Shanxi	Sichuan	Tianjin	Tibet	Xinjiang	Yunnan	Zhejiang
Harmonic mean of arithmetic means		2.77 (2.06–3.93)	1.68 (1.41–2.36)	2.21 (1.91–2.78)	2.12 (1.67–3.00)	1.79 (1.40–2.65)	2.75 (2.10–3.89)	Not applied	3.09 (2.12–4.89)	2.10 (1.42–2.78)	1.90 (1.59–2.55)
Harmonic mean of harmonic means		2.03 (1.27–2.93)	0.48 (0.30–1.11)	0.82 (0.40–1.83)	1.26 (0.68–2.60)	0.96 (0.62–1.73)	1.67 (0.78–3.38)	Not applied	1.98 (0.80–4.69)	1.28 (0.80–1.93)	1.23 (0.77–1.72)
Times doubled	1	1.33	2.05	0.28	1.20	0.66	1.00		2.00	2	1.00
	2	2.24	0.1	0.57	0.4	0.64	2		1.6	0.66	0.58
	3	3.76	0.19	1.15	1.06	0.77	2.22		3.06	0.84	1.23
	4		0.41	1.92	1.76	1.18	4.78		5.34	1.12	1.61
	5		0.86	2.92	2.2	1.55	3.57			1.61	2.29
	6		1.43	3.16	4.18	2.78				1.45	1.47
	7		2.66	6.13		4.49				7.32	3.48
	8		4.71								

Appendix Table 7. Sensitivity analysis 2: doubling times of COVID-19 cumulative incidence and their harmonic mean of the arithmetic means of the doubling times and harmonic mean of the harmonic means of the doubling times (95% CI) by province in mainland China, January 23–February 9, 2020.

Category		Mainland China (except Hubei)	Hubei	Anhui	Beijing	Chongqing	Fujian	Gansu	Guangdong	Guangxi	Guizhou	Hainan
Harmonic mean of arithmetic means		2.9 (2.62–3.24)	2.46 (2.37–2.55)	2.54 (2.12–2.99)	3.46 (2.77–4.57)	3.11 (2.38–4.17)	2.03 (1.29–3.10)	2.54 (1.80–3.89)	2.91 (2.40–3.61)	3.26 (2.37–4.22)	2.67 (1.85–3.92)	3.43 (2.57–4.62)
Harmonic mean of harmonic means		1.98 (1.82–2.17)	2.25 (2.18–2.33)	1.47 (0.90–2.29)	3.03 (2.23–3.99)	1.87 (1.28–2.80)	1.26 (0.69–2.01)	1.27 (0.66–2.84)	2.65 (2.14–3.10)	2.23 (1.33–3.29)	1.73 (0.67–3.40)	2.31 (1.40–3.71)
Times doubled	1	1.01	2.12	0.62	2.15	0.90	1.00	1.00	2.16	1.30	2.50	1.55
	2	1.59	1.47	1.38	3.50	2.04	1.11	1.14	2.29	2.84	3.50	2.28
	3	2.30	2.22	2.30	4.21	4.37	1.09	1.26	2.79	4.22	1.64	5.31
	4	3.21	3.03	3.74		7.99	1.71	4.10	4.45	8.50	2.56	6.86
	5	6.41	3.45	3.96			4.13	5.90			5.80	
		Hebei	Heilongjiang	Henan	Hunan	Inner Mongolia	Jiangsu	Jiangxi	Jilin	Liaoning	Ningxia	Qinghai
Harmonic mean of arithmetic means		1.91 (1.42–2.83)	2.21 (1.74–2.81)	1.87 (1.50–2.40)	1.89 (1.48–2.77)	2.39 (1.84–4.00)	2.31 (1.80–3.10)	1.89 (1.44–2.52)	3.01 (2.14–4.06)	2.44 (1.49–4.00)	2.68 (1.70–4.50)	3.21 (2.25–5.67)
Harmonic mean of harmonic means		0.81 (0.39–1.78)	1.47 (0.77–2.51)	0.85 (0.48–1.37)	0.75 (0.41–1.27)	1.16 (0.66–3.02)	1.60 (1.06–2.36)	1.18 (0.62–1.86)	2.44 (1.29–3.73)	0.99 (0.37–2.46)	1.88 (0.94–3.96)	1.8 (0.80–5.08)
Times doubled	1	0.33	0.80	0.39	0.26	1.00	1.00	0.63	3.00	0.50	2.00	2.33
	2	0.67	1.36	0.68	0.53	0.40	1.31	0.92	2.59	1.07	1.25	0.67
	3	1.60	2.12	0.71	1.30	0.85	1.75	1.78	3.53	2.76	2.55	4.00
	4	1.33	2.95	1.62	1.92	2.75	2.32	1.72	2.38	4.67	4.53	4.50
	5	2.01	3.04	2.73	2.19	4.71	4.07	1.74				
	6	5.28	3.31	3.96	4.56			3.88				
		Shaanxi	Shandong	Shanghai	Shanxi	Sichuan	Tianjin	Tibet	Xinjiang	Yunnan	Zhejiang	
Harmonic mean of arithmetic means		3.44 (2.76–4.40)	1.43 (1.18–2.14)	3.08 (2.52–4.07)	1.93 (1.50–3.09)	2.61 (1.89–3.60)	3.17 (2.16–4.59)	Not applied	3.05 (2.10–4.67)	1.82 (1.20–2.87)	2.37 (1.87–3.14)	

Category		Mainland China (except Hubei)	Hubei	Anhui	Beijing	Chongqing	Fujian	Gansu	Guangdong	Guangxi	Guizhou	Hainan
Harmonic mean of harmonic means		2.84 (1.82–4.05)	0.24 (0.14–0.60)	2.61 (1.72–3.59)	0.71 (0.32–1.88)	1.82 (1.28–2.57)	2.12 (0.79–4.34)	Not applied	1.86 (0.83–4.40)	1.03 (0.56–1.77)	1.98 (1.73–2.41)	
Times doubled	1	3.33	0.05	2.00	0.20	1.12	2.00		2.00	0.66	1.57	
	2	2.24	0.10	3.00	0.40	1.51	1.66		1.60	0.84	2.4	
	3	3.76	0.20	3.29	1.06	2.72	4.95		3.06	1.12	1.39	
	4		0.40		1.76	4.04	5.30		5.34	1.61	4.06	
	5		0.86		2.20					1.45		
	6		1.43		4.18					7.32		
	7		2.66									
	8		4.71									

Appendix Table 8. Websites of national and provincial health commissions in mainland China.*

Health commission	URL	Notes
National Health Commission of the People's Republic of China	http://www.nhc.gov.cn	
Provincial health commissions		
Anhui	http://wjw.ah.gov.cn	
Beijing	http://wjw.beijing.gov.cn	
Chongqing	http://wsjkw.cq.gov.cn	
Fujian	http://fjwsjk.fjsen.com	
Gansu	http://wsjk.gansu.gov.cn	
Guangdong	http://wsjkw.gd.gov.cn	
Guangxi	http://wsjkw.gxzf.gov.cn	
Guizhou	http://www.gzhfpc.gov.cn	
Hainan	http://wst.hainan.gov.cn	
Hebei	http://www.hebwst.gov.cn	Our team members found it often inaccessible from Statesboro, GA, USA.
Heilongjiang	http://wsjkw.hlj.gov.cn	
Henan	http://www.hnwsjsw.gov.cn	
Hubei	http://wjw.hubei.gov.cn	
Hunan	http://wjw.hunan.gov.cn	
Inner Mongolia	http://wjw.nmg.gov.cn	
Jiangsu	http://wjw.jiangsu.gov.cn	
Jiangxi	http://hc.jiangxi.gov.cn	
Jilin	http://www.jl.gov.cn	
Liaoning	http://www.shenyang.gov.cn	
Ningxia	http://wsjkw.nx.gov.cn/index.htm	
Qinghai	https://wsjkw.qinghai.gov.cn	
Shaanxi	http://sxwjw.shaanxi.gov.cn	
Shandong	http://wsjkw.shandong.gov.cn	Our team members found it persistently inaccessible from Statesboro, GA, USA.
Shanghai	http://www.shanghai.gov.cn	
Shanxi	http://wjw.shanxi.gov.cn	
Sichuan	http://wsjkw.sc.gov.cn	
Tianjin	http://www.tj.gov.cn	
Tibet	http://wjw.xizang.gov.cn/	
Xinjiang	http://www.xjhfpc.gov.cn/	
Yunnan	http://ynswsjkw.yn.gov.cn/	
Zhejiang	https://www.zjwjw.gov.cn	

* If our team was unable to directly retrieve the press release from a provincial health commissions, we used mainland Chinese media reports that directly reported on the provincial health commissions' announcements. Note that mainland Chinese media are controlled by the Chinese Communist Party and they could not deviate from the government's announcements.

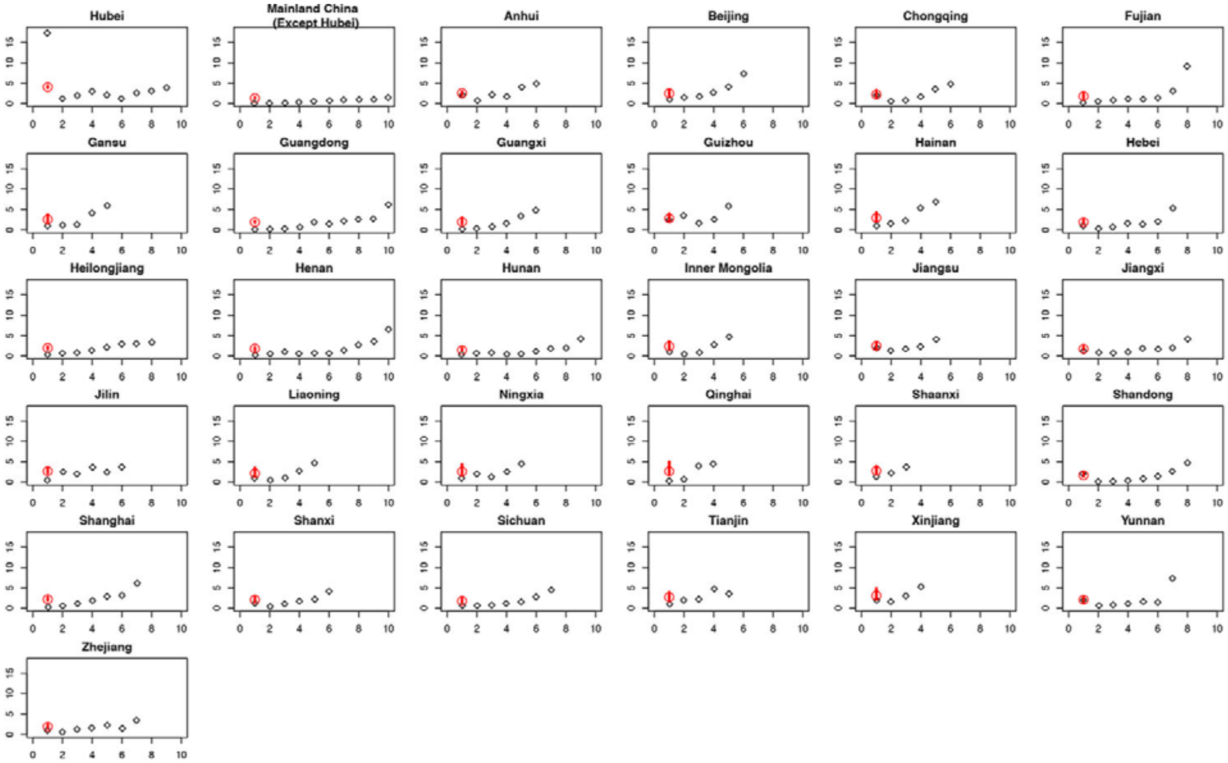

Appendix Figure 1. Main analysis: The harmonic mean of the arithmetic means of COVID-19 epidemic doubling times (red circles) with 95% confidence interval (red bars) of the doubling times (days), and their values (black diamonds) by the number of times the reported cumulative incidence doubled by province within mainland China, January 20–February 9, 2020. Each panel represents a province except the panel labeled “Mainland China (except Hubei),” which is the aggregate of all other provinces in mainland China, except Hubei. Doubling time for Tibet is not available, because there had been only 1 confirmed case in Tibet as of February 9, 2020. The *x*-axis represents the *n*th time the reported cumulative incidence doubled and the *y*-axis represents the value of the doubling times.

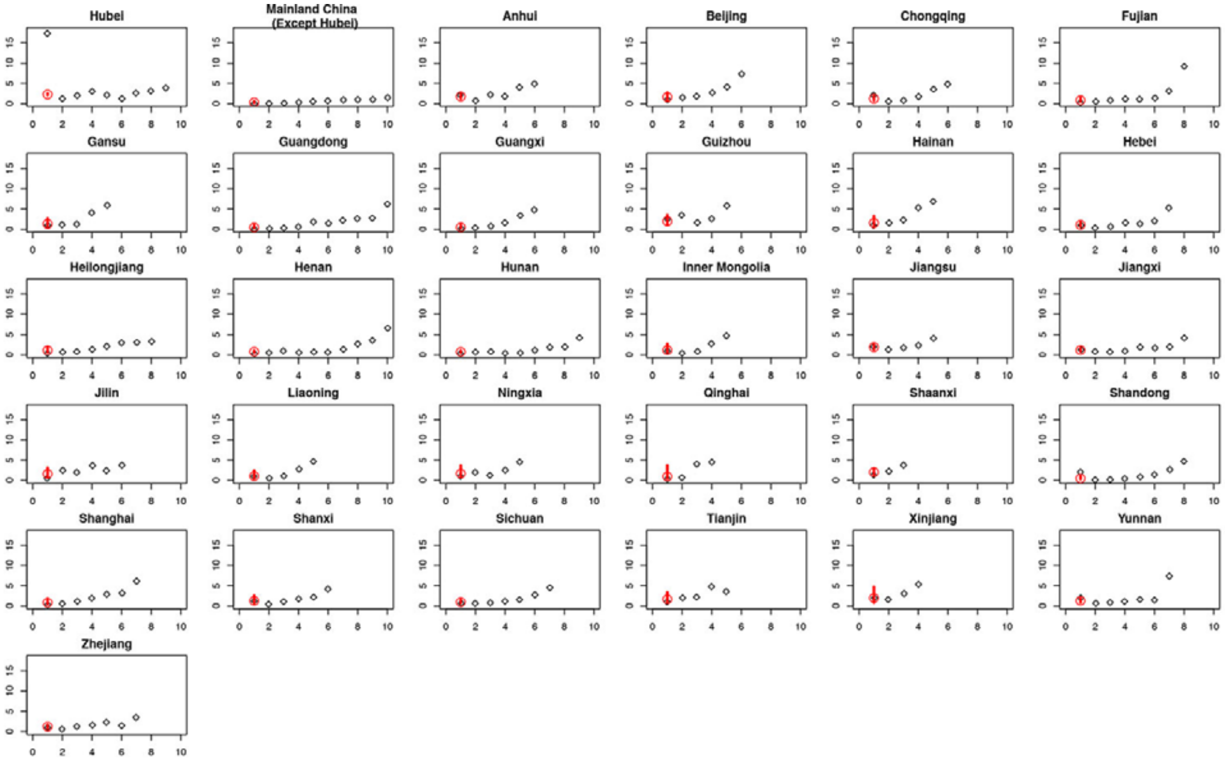

Appendix Figure 2. Main analysis: The harmonic mean of the harmonic means of COVID-19 epidemic doubling times (red circles) with 95% confidence interval (red bars) of the doubling times (days), and their values (black diamonds) by the number of times the reported cumulative incidence doubles by province within mainland China, from January 20–February 9, 2020. Each panel represents a province except the panel labeled “Mainland China (except Hubei),” which is the aggregate of all other provinces in mainland China, except Hubei. Doubling time for Tibet is not available, because there had been only 1 confirmed case in Tibet as of February 9, 2020. The *x*-axis represents the *n*th time the reported cumulative incidence doubled and the *y*-axis represents the value of the doubling times.

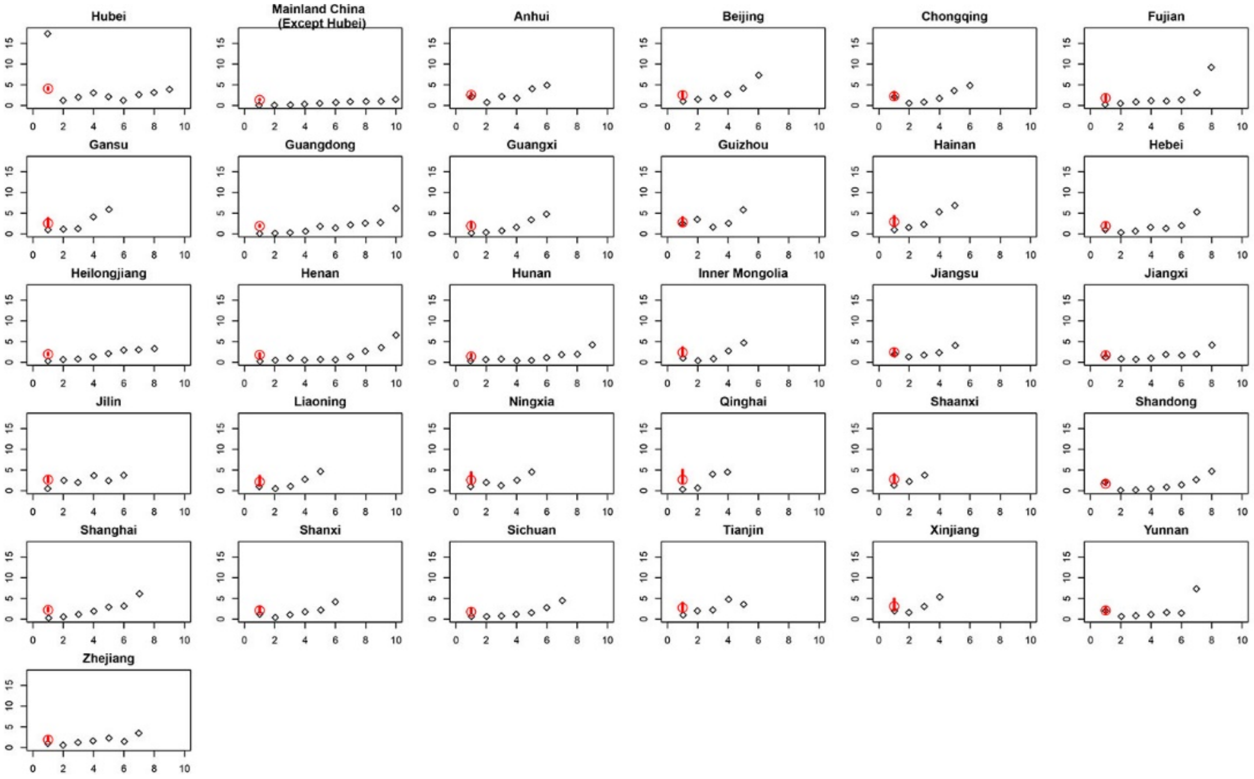

Appendix Figure 3. Sensitivity analysis 1: The harmonic mean of the arithmetic means of COVID-19 doubling times (red circles) with 95% confidence interval (red bars) of the doubling times (days), and their values (black diamonds) by the number of times the reported cumulative incidence doubled by province within mainland China, December 31, 2019–February 9, 2020. Each panel represents a province except the panel labeled “Mainland China (except Hubei),” which is the aggregate of all other provinces in mainland China, except Hubei. Doubling time for Tibet is not available, because there had been only 1 confirmed case in Tibet as of February 9, 2020. The *x*-axis represents the *n*th time the reported cumulative incidence doubled and the *y*-axis represents the value of the doubling times.

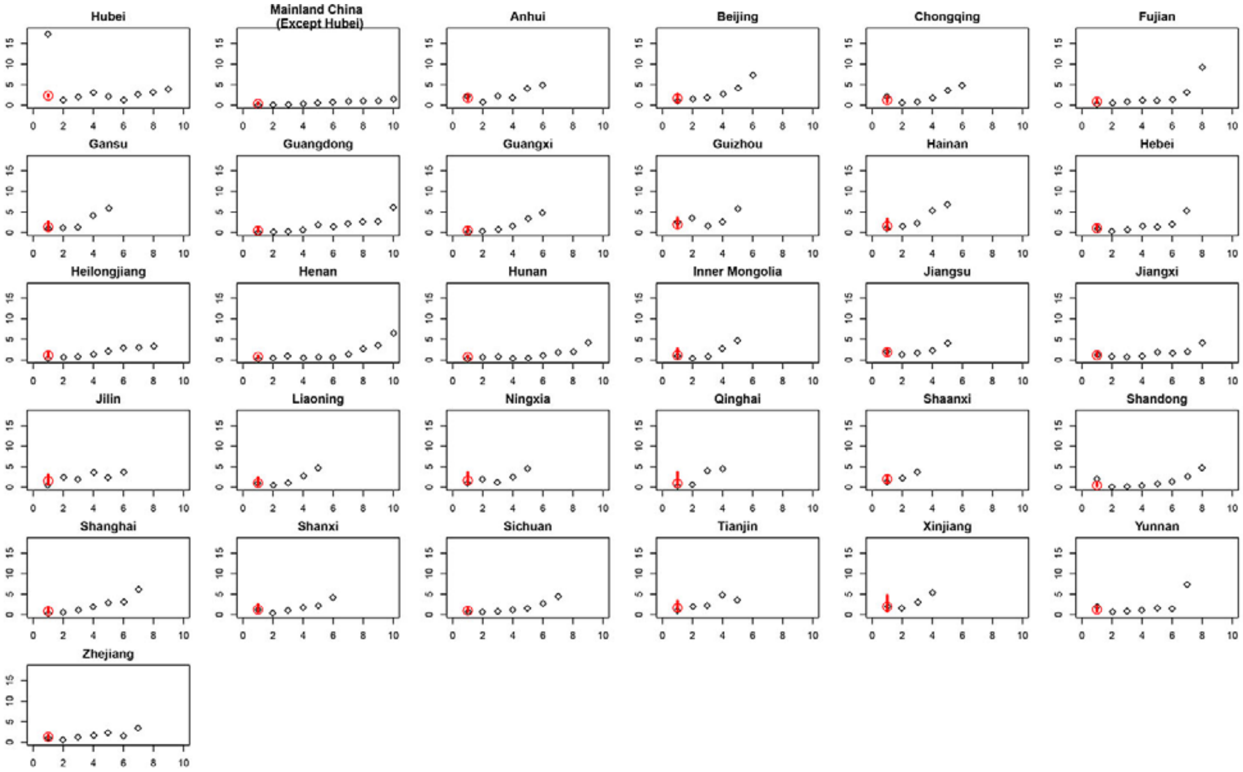

Appendix Figure 4. Sensitivity analysis 1**:** The harmonic mean of the harmonic means of COVID-19 doubling times (red circles) with 95% confidence interval (red bars) of the doubling times (days), and their values (black diamonds) by the number of times the reported cumulative incidence doubled by province within mainland China, December 31, 2019–February 9, 2020. Each panel represents a province except the panel representing "Mainland China (except Hubei)" that is the aggregate of all other provinces in mainland China, except Hubei. Doubling time for Tibet is not available, because there had only been 1 confirmed case in Tibet as of February 9, 2020. The *x*-axis represents the *n*th time the reported cumulative incidence doubled and the *y*-axis represents the value of the doubling times.

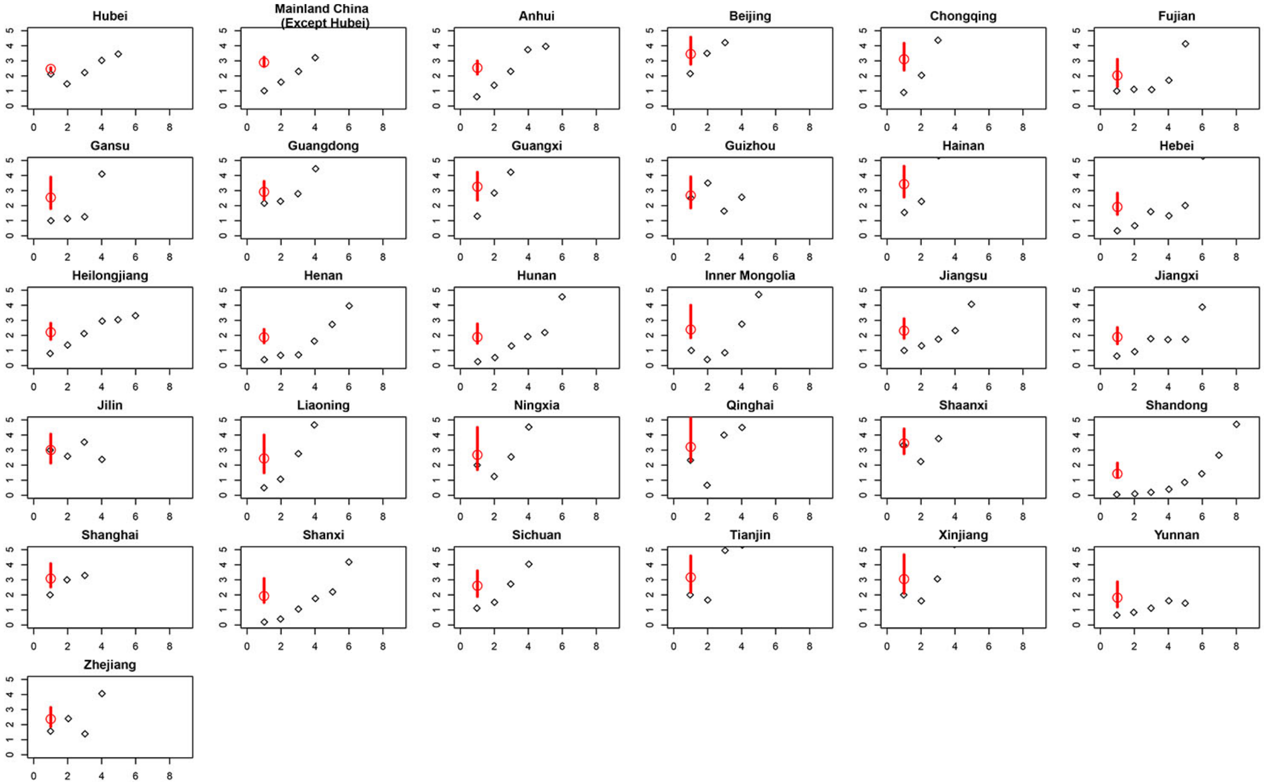

Appendix Figure 5. Sensitivity analysis 2: The harmonic mean of the arithmetic means of COVID-19 doubling times (red circles) with 95% confidence interval (red bars) of the doubling times (days), and their values (black diamonds) by the number of times the reported cumulative incidence doubles by province within mainland China, January 23–February 9, 2020. Each panel represents a province except the panel labeled "Mainland China (except Hubei)," which is the aggregate of all other provinces in mainland China, except Hubei. Doubling time for Tibet is not available, because there had been only 1 confirmed case in Tibet as of February 9, 2020. The *x*-axis represents the *n*th time the reported cumulative incidence doubled and the *y*-axis represents the value of the doubling times.

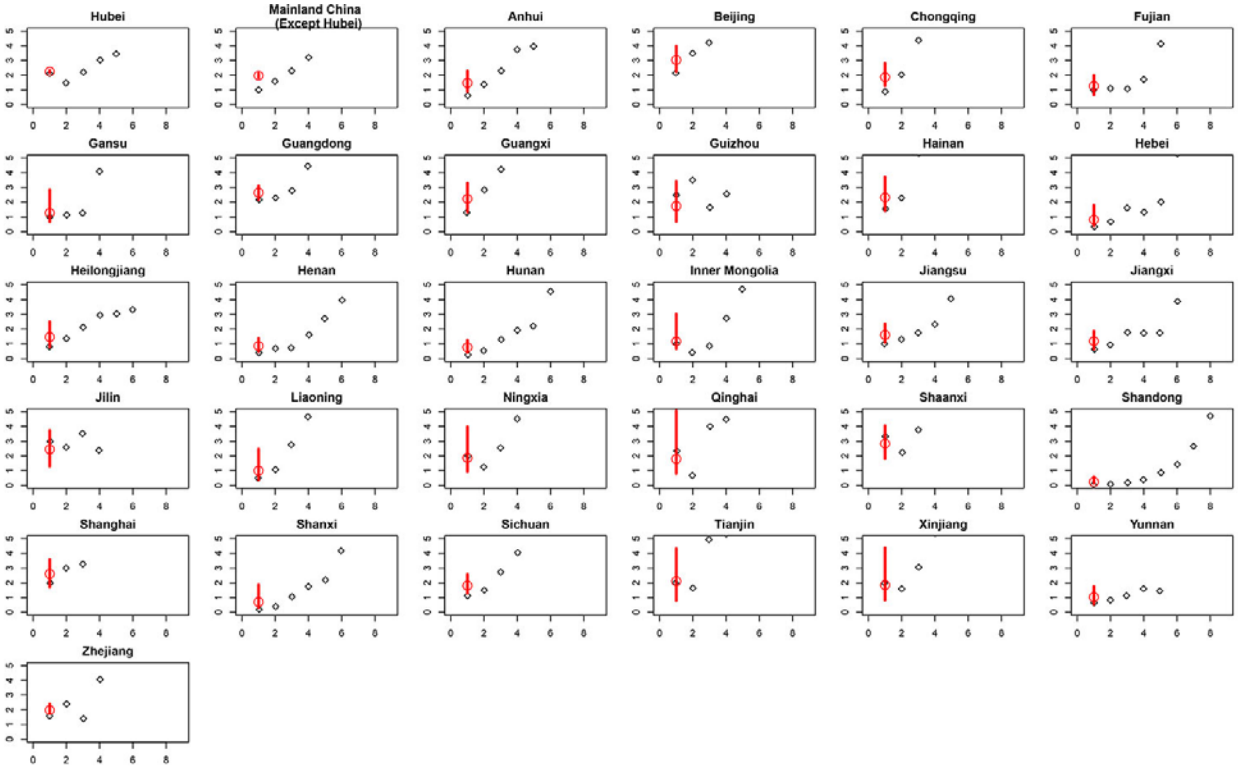

Appendix Figure 6. Sensitivity analysis 2: The harmonic mean of the harmonic means of COVID-19 doubling times (red circles) with 95% confidence interval (red bars) of the doubling times (days), and their values (black diamonds) by the number of times the reported cumulative incidence doubles by province within mainland China, January 23–February 9, 2020. Each panel represents a province except the panel labeled "Mainland China (except Hubei)," which is the aggregate of all other provinces in mainland China, except Hubei. Doubling time for Tibet is not available, because there had been only 1 confirmed case in Tibet as of February 9, 2020. The *x*-axis represents the *n*th time the reported cumulative incidence doubled and the *y*-axis represents the value of the doubling times.

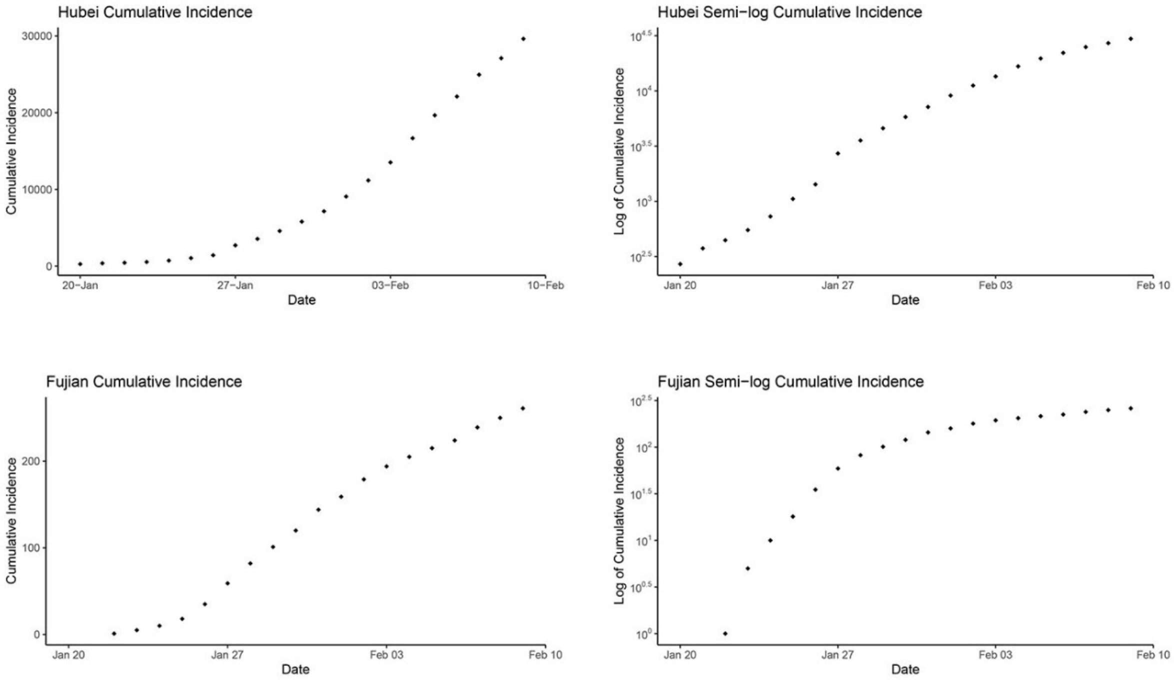

Appendix Figure 7. Cumulative incidence and $\log_{10}$ cumulative incidence over time (date) for Hubei (upper panel) and Fujian (lower panel).

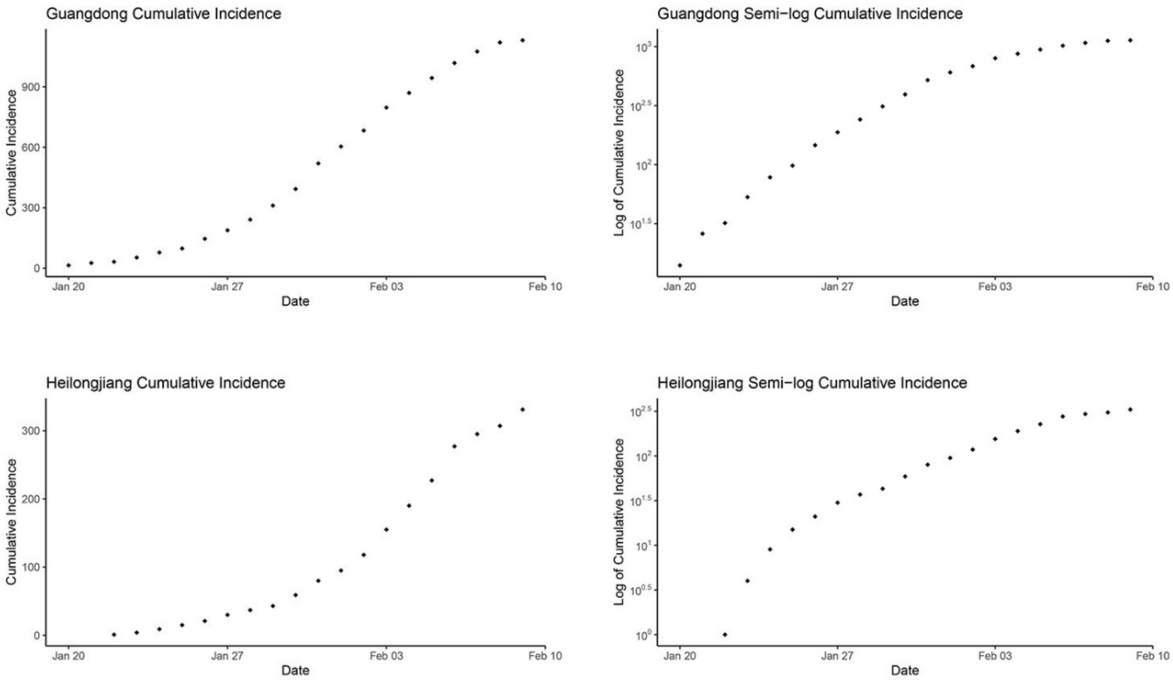

Appendix Figure 8. Cumulative incidence and $\log_{10}$ cumulative incidence over time (date) for Guangdong (upper panel) and Heilongjiang (lower panel).

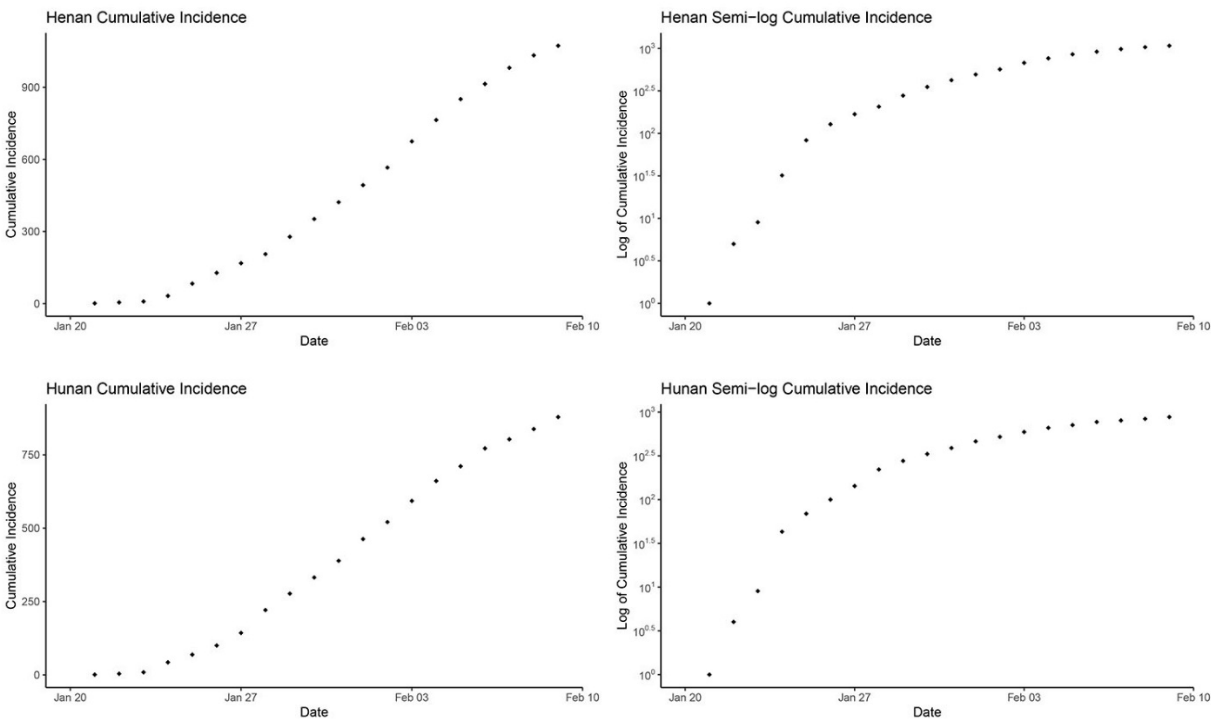

Appendix Figure 9. Cumulative incidence and $\log_{10}$ cumulative incidence over time (date) for Henan (upper panel) and Hunan (lower panel).

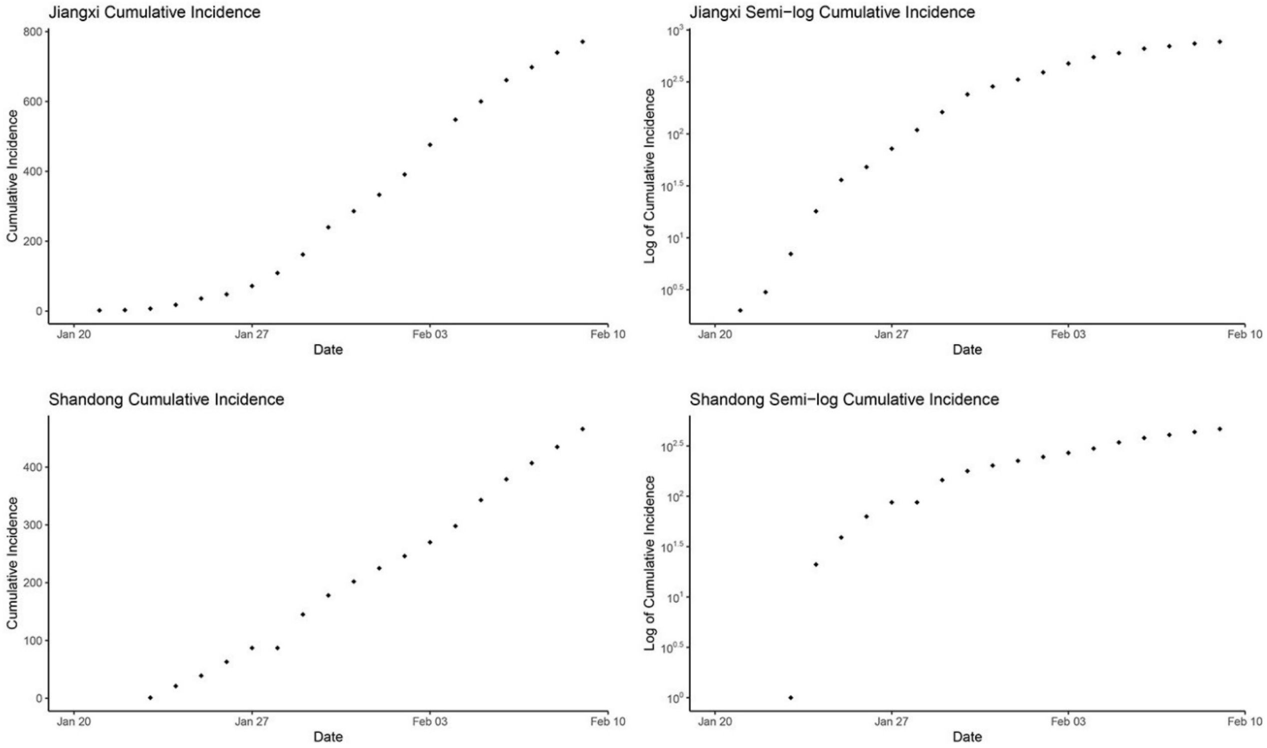

Appendix Figure 10. Cumulative incidence and $\log_{10}$ cumulative incidence over time (date) for Jiangxi (upper panel) and Shandong (lower panel).

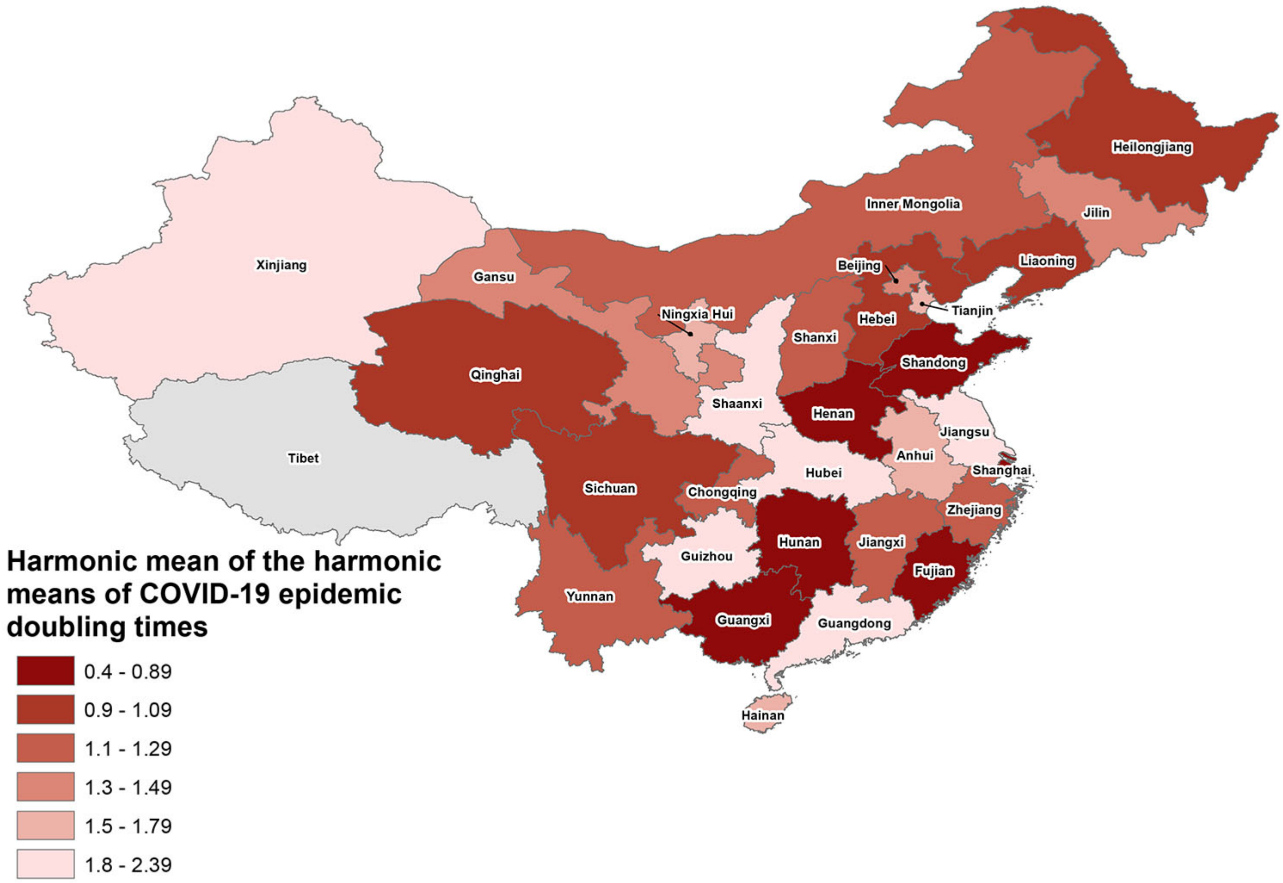

Appendix Figure 11. Main analysis: Map of the harmonic mean of the harmonic means of COVID-19 by province in mainland China, January 20–February 9, 2020.

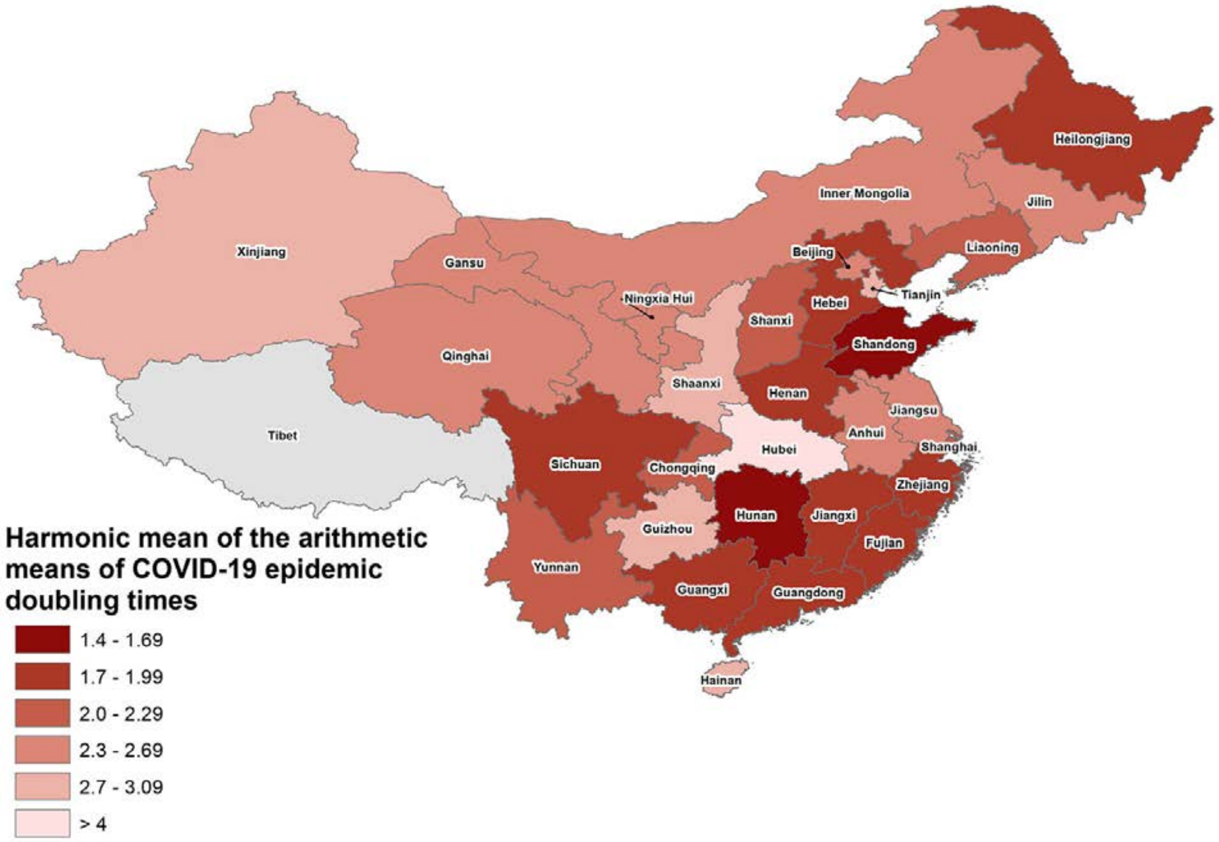

Appendix Figure 12. Sensitivity analysis 1: Map of the harmonic mean of the arithmetic means of COVID-19 by province in mainland China, December 31, 2019–February 9, 2020.

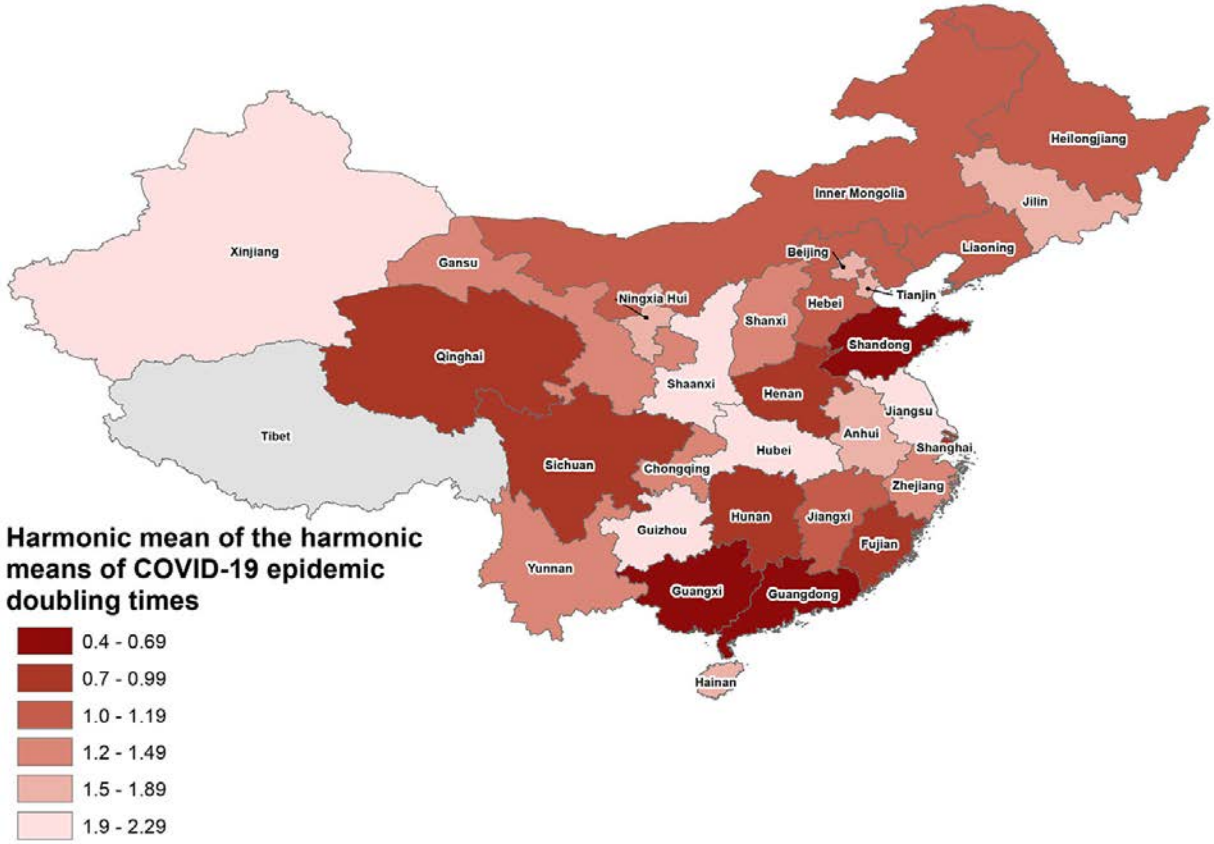

Appendix Figure 13. Sensitivity analysis 1: Map of the harmonic mean of the harmonic means of COVID-19 by province in mainland China, December 31, 2019–February 9, 2020.

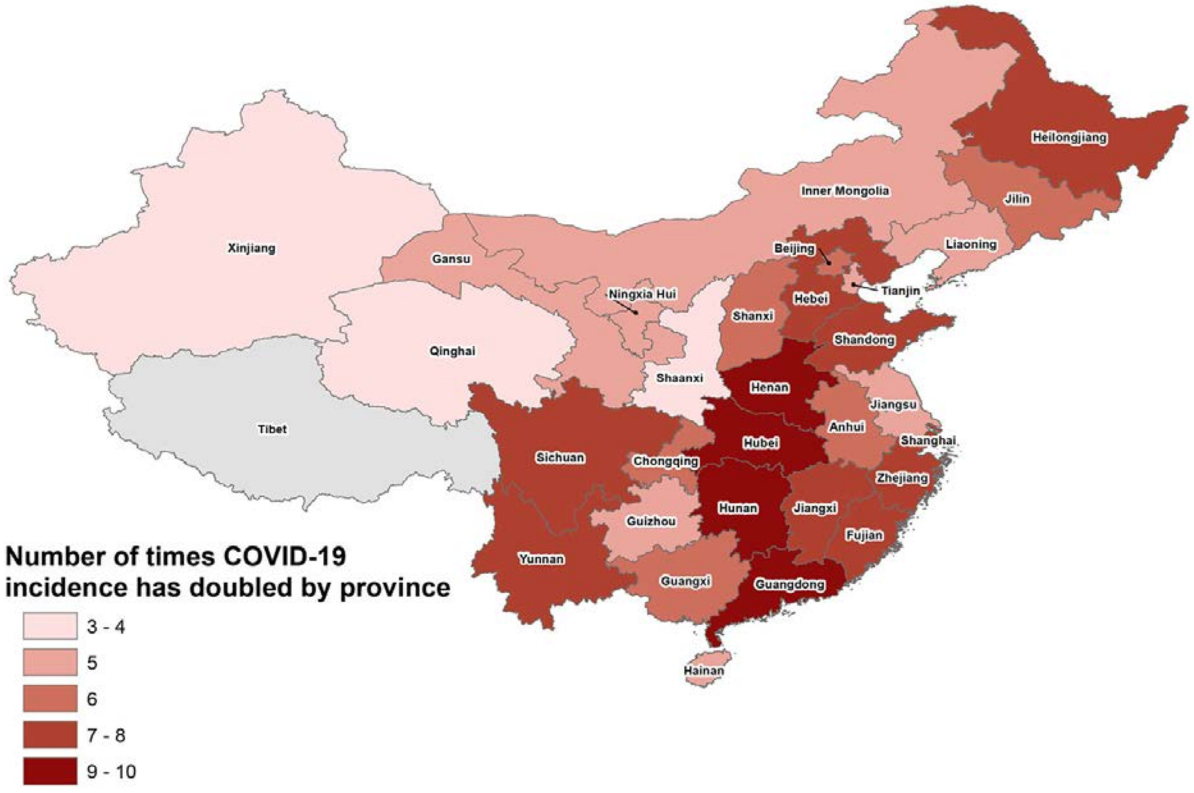

Appendix Figure 14. Sensitivity analysis 1: Map of the number of times the COVID-19 outbreak has doubled by province in mainland China, December 31, 2019–February 9, 2020.
